# A Mammalian Cell Based FACS-Panning Platform for the Selection of HIV-1 Envelopes for Vaccine Development

**DOI:** 10.1371/journal.pone.0109196

**Published:** 2014-10-03

**Authors:** Tim-Henrik Bruun, Katharina Mühlbauer, Thomas Benen, Alexander Kliche, Ralf Wagner

**Affiliations:** Institute of Medical Microbiology and Hygiene, University Regensburg, Regensburg, Bavaria, Germany; Duke University Medical Center, United States of America

## Abstract

An increasing number of broadly neutralizing monoclonal antibodies (bnMAb) against the HIV-1 envelope (Env) protein has been discovered recently. Despite this progress, vaccination efforts with the aim to re-elicit bnMAbs that provide protective immunity have failed so far. Herein, we describe the development of a mammalian cell based FACS-panning method in which bnMAbs are used as tools to select surface-exposed envelope variants according to their binding affinity. For that purpose, an HIV-1 derived lentiviral vector was developed to infect HEK293T cells at low multiplicity of infection (MOI) in order to link Env phenotype and genotype. For proof of principle, a gp145 Env model-library was established in which the complete V3 domain was substituted by five strain specific V3 loop sequences with known binding affinities to nMAb 447-52D, respectively. Env genes were recovered from selected cells by PCR, subcloned into a lentiviral vector (i) to determine and quantify the enrichment nMAb binders and (ii) to generate a new batch of transduction competent particles. After 2 selection cycles the Env variant with highest affinity was enriched 20-fold and represented 80% of the remaining Env population. Exploiting the recently described bnMAbs, this procedure might prove useful in selecting Env proteins from large Env libraries with the potential to elicit bnMAbs when used as vaccine candidates.

## Introduction

The HIV-1 envelope protein (Env) is translated as a 160 kDa precursor glycoprotein. gp160 is cleaved by a furin protease into an extracellular moiety gp120 and a transmembrane domain gp41. These non-covalently associated heterodimers form trimeric complexes exposed on the host cell membrane. Env is the only viral protein that is exposed on both the host cellular and viral membrane.

To date, almost all licensed vaccines against viral pathogens are believed to protect by inducing pathogen specific antibodies. Despite global efforts, the development of a vaccine that is capable of mediating an antibody based protective immunity against HIV has failed so far. During the past two decades, more than 30 candidate vaccines have been tested in human clinical trials [1], [2]. These studies assessed replicating or replication-defective vectored vaccines encoding selected HIV-1 antigens, HIV-1 DNA or RNA vaccines as well as soluble HIV-1 proteins and peptide derivatives, in various adjuvant formulations and prime/boost regimens [2]–[6]. To the extent, these efforts have been taken forward to phase IIB or phase III efficacy trials, strategies that have successfully worked for other pathogens have mostly failed to elicit protective immunity towards HIV-1 infection. Cautious optimism was created by the recently published results of the RV144 trial [3] that revealed a ∼30% protection in those volunteers, who received the vaccine. Notably, a non-neutralizing IgG antibody response against V1/V2 and in particular IgG3 specific antibodies seem to contribute to protection from infection [7].

Although it is widely agreed that an effective vaccine will need to induce both B-cell and T-cell (CD4^+^ and CD8^+^) responses [6], the exact mode needed for a protective, vaccine-induced immune response against HIV-1 is still unclear. Conceptually, an early neutralization of HIV-1 before an infection of target cells can occur, e.g. during mucosal transmission seems highly attractive in order to avoid integration of HIV-1 and formation of latently infected reservoirs [8]–[10]. Passive immunization experiments provided ample evidence that a vaccine, which is able to induce bnMAbs in sufficient concentrations at the mucosal entry sites can protect from infection [11], [12].

Recently, several “reverse vaccinology” [13] approaches that aimed at shifting the immune response [14] towards neutralization relevant Env epitopes led to promising results [15], [16]: Applying a directed molecular evolution approach, Du et al. [17] identified chimeric gp120 Env variants (ST-008), which elicited neutralizing antibody responses in rabbits. Other approaches intended to shift the immune response by heterologous substitutions or deletion of the V1 loop, thus improving the immunogenicity of several potentially beneficial epitopes [18]. Alternatively, targeted hyperglycosylation of variable loops [19] or chemical cross-linking [20], are used for focussing antibody responses to desired epitopes such as the CD4 binding site.

Recent evidence raised hope that trimeric Env complexes may have the potential to induce broadly neutralizing antibodies that targets highly potent neutralizing structures e.g. quaternary epitopes [17], [21]–[25]. Screening technologies, which allow the selection of trimeric Env out of a large library by bnMAb affinity-enrichment may therefore lead to the identification of Env complexes capable of re-eliciting antibody responses with broadened neutralization profiles [11], [15], [26]–[28].

Recently, several broadly neutralizing monoclonal antibodies (bnMAbs) have been discovered [1], [2], [11], [29], accelerating the antibody (B-cell) mediated vaccine strategies [2]–[6], [30]. Therefore, it has been hypothesized that particularly the presentation of trimeric Env complexes as also found on the virus or cell membrane may be necessary at least for the induction of some highly potent bnMAbs e.g. PG16 [3], [23], [31]. Herein, describe the development of a FACS-based, mammalian-cell display- and panning- platform in order to screen for such potential HIV-1 candidate vaccines. For proof-of-principle, a chimeric Env/V3 model-library of few variants with known affinities to a V3-binding antibody 447-52D [7], [32]–[36] was successfully established and used to demonstrate the intended enrichment of a membrane-bound HIV-1 Env/V3-variant which yielded highest affinity to the applied antibody.

## Results

### FACS-panning procedure

**Figure 1:**
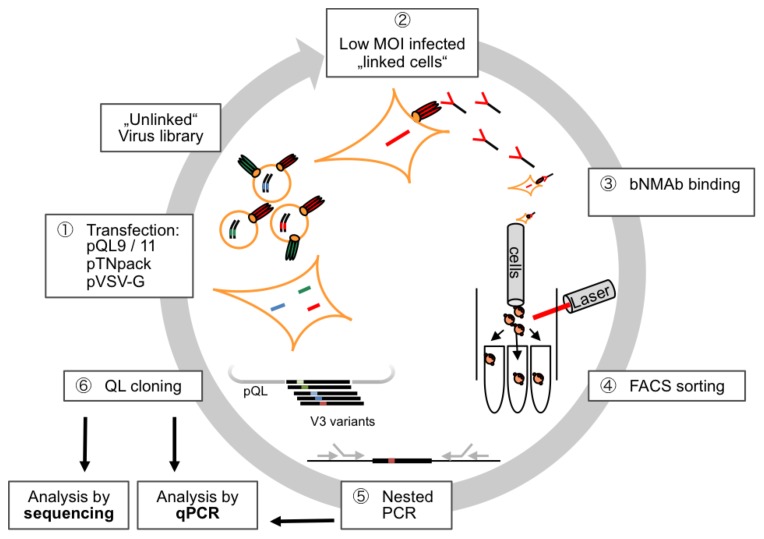
Schematic overview of the FACS-panning procedure. HEK293T cells are transfected with a plasmid mix comprising (i) a lentiviral vector plasmid (pQL9/11) encoding the Env/V3 model library, (ii) a packaging construct (pTNpack) as well as (iii) pVSV-G to render released particles transduction competent (1). Linkage between geno- and phenotype from the initially “unlinked” virus library is achieved by low MOI infection of new cells (2). Antibodies (e.g. bnMAb) are applied to bind cells expressing the various envelopes, respectively (3). Envelope expressing cells displaying the highest MFI to the bnMAb and an internal control (GFP) are selected by a FACS-sorting procedure (4). Cells are collected and lysed prior to amplifying the envelope genes from genomic DNA (5). The recovered envelope genes are cloned into pQL9/11 and analyzed by sequencing and realtime-PCR (6). Fresh cells are transfected (together with pTN pack and pVSV-G) to produce new virus for Low MOI infection of fresh cells, thus entering a new cycle of selection.

Herein, we describe the development of a mammalian-cell based surface display and antibody driven FACS-screening method that allows the identification of affinity enriched variants of the HIV-1 envelope protein. The ultimate goal is to provide a technology platform enabling the identification of HIV candidate vaccines from comprehensive envelope libraries. In brief, the procedure as developed comprises six steps, which are summarized in Figure 1 and will be described in the following sections in more detail.

### HIV-1 chimeric Env/V3 model library and 447-52D model antibody

**Figure 2:**
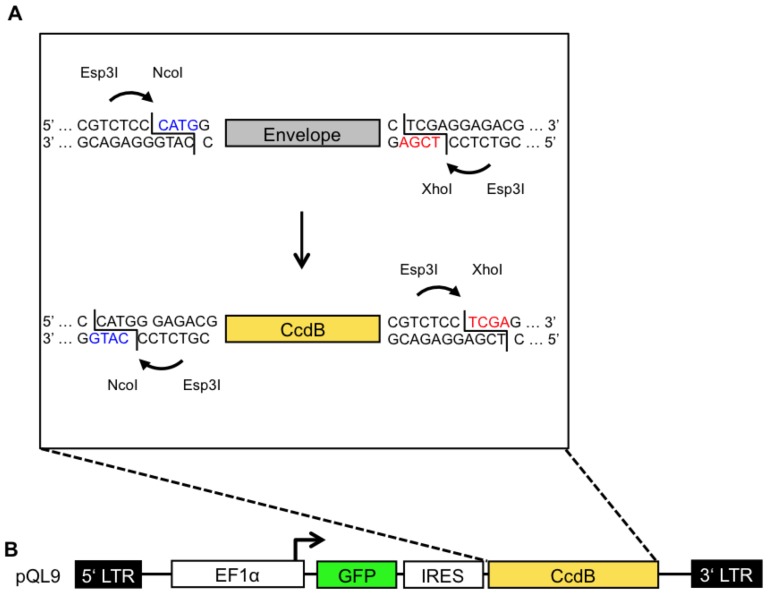
Schematic overview of the QL cloning procedure. An envelope gene or an envelope library is amplified with primers to introduce flanking Esp3I restriction sites enabling the generation of a 5′ NcoI and a 3′ Xho sitey (A; top). The envelope gene or an envelope library is incubated together with pQL9/11 in a one-tube reaction with Esp3I and T4-Ligase. Compatible “sticky-ends” (equally colored) can be ligated successfully, direct proper orientation and mediating resistance for further cleavage (A). Following transformation of CcdB sensitive bacteria, only recipients bearing a plasmid without CcdB are able to form colonies in the presence of ampicillin. (B) The lentiviral vector construct pQL9 comprises (i) 5′LTR (Long terminal repeat), (ii) EF1α (human promotor), (iii) GFP (marker gene), (iv) an IRES (internal ribosome entry site), (v) a CcdB positive selection marker [58], and (vi) a 3′LTR sequence.

As model antibody in the proof of concept study the neutralizing monoclonal antibody (nMAb) 447-52D [6], [32]–[34] was selected for the following reasons: (i) 447-52D was available at the onset of the study in sufficient amounts, (ii) distinct binding affinities to V3 loop peptides representing 5 different virus strains (MN, RF, CDC42, HXB2, SF33) were known [8]–[10], [34], (iii) 447-52D has been described as a neutralizing monoclonal antibody, shown to neutralize mainly clade-B Tier 1 and some Tier 2 isolates, targeting a linear (conformational influenced) epitope [11], [12], [30], [37]. For proof of principle, a gp145 Env model library comprising the external glycoprotein gp120, the extracellular gp41 domain as well as the gp41 derived transmembrane domain was established in which the complete V3 domain within a clade C 96ZM Env backbone was substituted by the above strain specific V3 loop sequences, respectively. The resulting chimeras were cloned into the lentiviral pQL vector system to generate the 96ZM Env/V3 model library (Figure 2).

### QL-cloning system with pQL plasmids

For convenient and highly efficient cloning of prospectively larger Env libraries the design of lentiviral vector constructs was optimized to enable a one-tube QL (Quick-Ligation) restriction-ligation procedure modified from the “golden-gate” system [13], [38], [39] (Figure 2 A).

With this system usually only very few (<0.01%) false positive bacteria were obtained on control plates. Consequently, almost all bacterial colonies, as probed by PCR, were successfully transformed with an Env coding plasmid (>99,99%) as intended.

### Expression of chimeric Env/V3 variants correlates with reporter GFP expression

**Figure 3:**
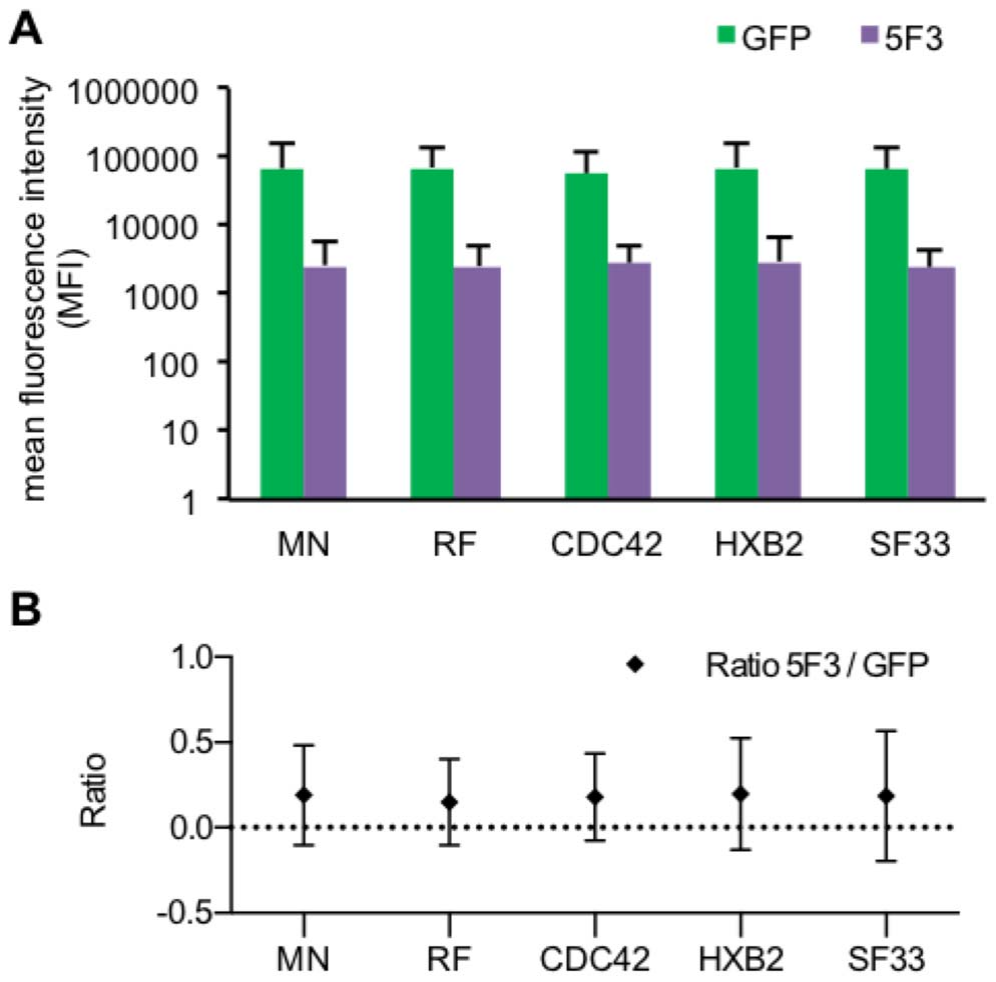
Expression levels of chimeric Env/V3 and GFP. HEK293T (3×10^5^) cells were separately transduced at MOI 1 by one of the pQL9 derived transduction competent particles encoding one of the chimeric Env/V3-variants, respectively. 72 h after transduction, cells were harvested and stained with an APC-labeled 5F3 antibody binding a constant region within the extracellular gp41 mojety and compared to GFP mediated fluorescence (Pearson correlation p<0,01 for each variant). **A** FACS analyses are depicted as the mean fluorescence intensity (MFI) of APC labeled 5F3 antibody- and GFP-signals for all chimeric Env/V3 variants, respectively. **B** The MFI ratios of 5F3 to GFP signals were calculated, respectively.

Expression of the chimeric Env variants within the Env/V3 library was genetically linked to GFP expression via an IRES site, respectively (Figure 2 B). Equal expression of each chimeric Env/V3 variant in relation to GFP was observed after staining of transfected cells with an antibody (5F3), which was selected due to its binding capabilities to a constant region shared by all chimeric Env variants of the model library, respectively (Figure 3 A).

GFP and Env expression correlated positively (Pearson correlation p<0.01) for all chimeric Env/V3 variants tested, respectively, (Figure 3 B, Figure S3), thus nicely confirming translational coupling the GFP marker and envelope expression.

### Affinity analysis of 447-52D antibody binding to individual members of the chimeric Env model library

**Figure 4:**
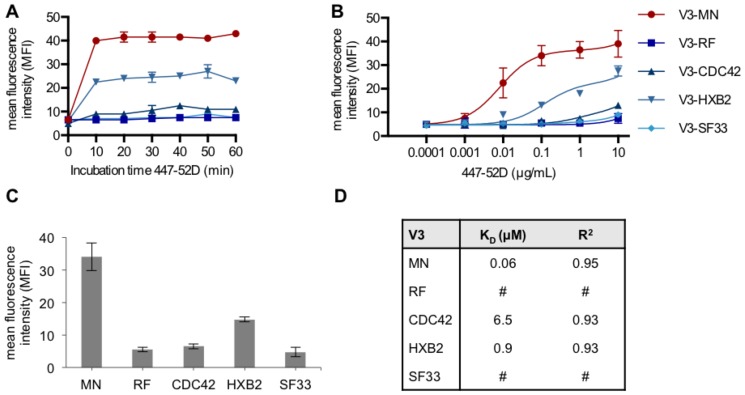
Affinity analysis of 447-52D antibody to individual members of the Env/V3 library. HEK293T cells were separately transduced at low MOI (0.1) by one of the pQL9 derived lentiviral particles expressing the indicated chimeric Env/V3-variant, respectively. 48 h post transduction, cells were stained with MAb 447-52D and an APC-labeled secondary antibody. The mean values of two independent experiments corrected for equal GFP expression are shown. **A** A time-course with 447-52D antibody (10 µg/mL) was performed to record the incubation-time needed for equilibrium binding. FACS data are expressed as the MFI for every member of the model library at the different incubation times, respectively. **B** 447-52D antibody concentrations were serially diluted and incubated on infected cells at equilibrium incubation-time (1 h at 4°C) to obtain a concentration dependent binding profile. **C** Cell populations stained with 0.1 µg/mL 447-52D antibodies are depicted separately in order to highlight the differential binding at this concentration. **D** Dissociation constants (K_D_) were calculated from the antibody-titration curves shown in (B) based on the µM concentrations derived from the 447-52D molecular weight and therefore expressed as Kd (µM). Data points were fitted by nonlinear least squares regression (One site; Fit total and nonspecific binding, Graphpad Prism 5), and the resulting K_d_ as well as R^2^ values are listed (#, non-calculable).

As a prerequisite for calculating the affinity of antibody 447-52D to each member of the chimeric Env/V3 library a time-course was performed (Fig. 4A) and the impact of various antibody dilutions were determined (Fig. 4B). For this purpose, equal numbers of HEK293T cells were transduced separately, each at a low MOI (0.1), respectively (Figure 4).

Incubation times longer than 10 min. did not lead to higher MFI, respectively (Figure 4 A). An optimal antibody concentration of 0.2 µg/mL was calculated to achieve the best discrimination between the various chimeric Env variants (Figure 4 B/C). The chimeric Env/V3 variant-MN showed highest affinity, followed by variant-HXB2, -CDC42. Due to low signal intensity we were unable to calculate Kd values for variant RF and SF33 (Figure 4 D). As a result, the demonstrated distribution of affinities was considered feasible for the purpose of evaluating the following panning procedure.

### The FACS-panning strategy

**Figure 5:**
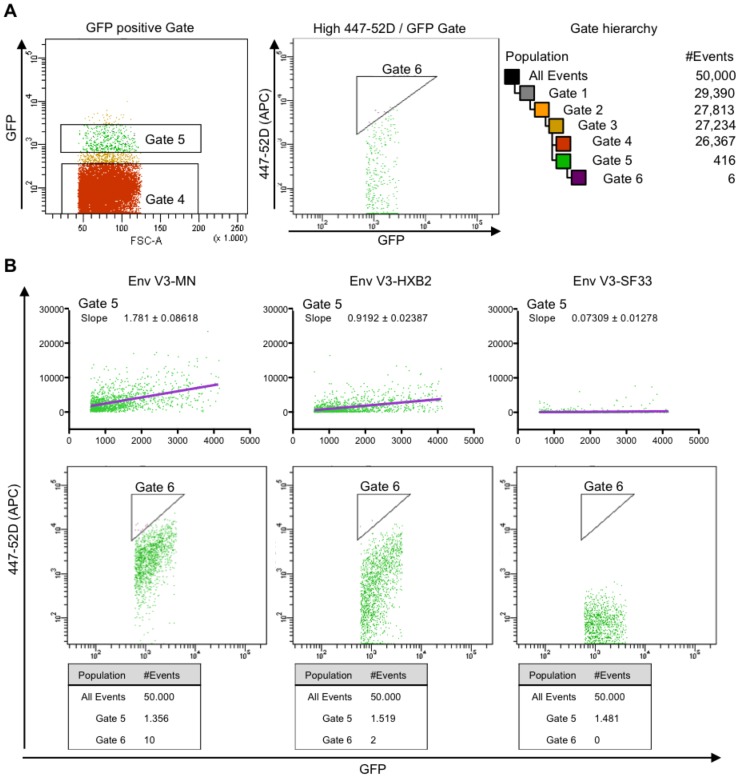
FACS-panning: gating strategy. HEK293T (13×10^6^) cells were transduced at low MOI (0.1) (**A**) by lentivirus particles derived from a plasmid mixture containing equal amounts of all Env/V3 variants and (**B**) by separately lentiviral particles each encoding only one of the chimeric Env/V3-variants, respectively. Equal amounts of transduced cells were harvested 72 h post transduction and were analysed by FACS. **A** One typical sorting experiment is shown to illustrate the FACS gating and sorting strategy: 50,000 events were recorded and a series of hierarchical gates were applied to isolate single cells. GFP positive cells were gated within their main population (Gate 5) to exclude multiply infected cells (high GFP) and background noise (low GFP). The GFP main population was subsequently gated for strong 447-52D antibody binding (high APC) in relation to Env expression (coupled to the GFP signal). Therefore, a triangle shaped Gate (Gate 6) was chosen. The complete Gate hierarchy starting with all events is illustrated. **B** Evaluation of the triangle-gate strategy: HEK293T cells (13×10^6^) were transduced at low MOI (0.1) with each pQL9 Env/V3-virus variant, respectively. Cells were stained with 447-52D antibody and analyzed by FACS according to the gating strategy described in A. Cells that appeared in Gate 5 were further analyzed by calculating a linear regression curve (purple). Slope values calculated for representative variants Env/V3-MN, Env/V3-HXB2, and Env/V3-SF33 are indicated. The triangle shaped Gate 6 is shown in a log-scale dot plot. The numbers of events counted for Gate 5 and Gate 6 are shown below each plot, respectively. Decreasing amounts of cells equal decreasing affinities of Env, as detected in the sorting Gate 6, ranging from 10 counts of Env/V3-MN, 2 of Env/V3-HXB2 and 0 of Env/V3-SF33, respectively.

In order to elaborate - defined conditions – for a feasible FACS based gating strategy with optimal selection performance, HEK293T (13×10^6^) cells were transduced at low MOI (0.1), 1^st^ by lentivirus particles derived from a plasmid mixture containing equal amounts of all Env/V3 variants (Fig. 5A) and 2^nd^ by separately lentiviral particles each encoding only one of the chimeric Env/V3-variants, respectively (Fig. 5B). Equal amounts of transduced cells were harvested 72 h post transduction and were analysed by FACS (Figure 5).

A typical sorting experiment is shown to illustrate the resulting conditions for FACS-panning strategy. In order to select for living, single cells a commonly used hierarchically gating strategy was chosen (Figure 5 A). Cells infected with separate virus stocks of each of the chimeric Env/V3-variants were applied to FACS-sorting procedures utilizing a triangle shaped Gate (Gate 6, Figure 5 B) for selection, respectively. Subsequently, data were analyzed and a linear regression curve was calculated for each variant. For clarity reasons, results are only shown for Env variants-MN, -HXB2 and -SF33, representing a good (MN), intermediate (HXB2) and weak (SF33) 447-52D binder (Figure 5 B). The distinct slopes of the regression curves demonstrate the different ratio of 447-52D/GFP signals for each Env variant, respectively. A higher ratio equals an higher affinity of 447-52D towards the respective Env [14], [40]. Therefore, stringency in selecting high affinity Envs was achieved by gating only the top 0.005% of all events (Figure 5 B, MN) [15], [16], [40], [41]. The distribution of cells reached with these parameters suggested that only cells expressing Env variant-MN (10-counts) or -HXB2 (2-counts) would be selected using the described FACS-sorting protocol (Figure 5 B, Gate 6), leading to a stringent discrimination of binder versus non-binder Env/V3-variants.

### FACS-panning: Evaluation screen

**Figure 6:**
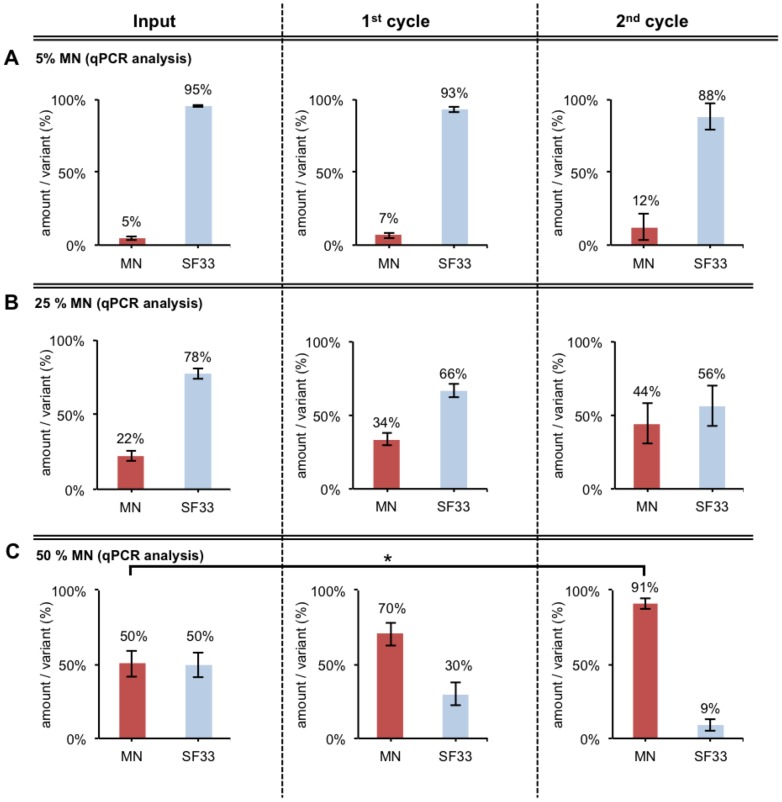
FACS-panning using different ratios of Env/V3-variants MN and SF33. Different pQL9 Env/V3-variant plasmid mixtures containing **A** 5% MN/95% SF33, **B** 25% MN/75% SF33, **C** 50% MN/50% SF33 were prepared. HEK293T cells (13×10^6^) were transfected (pQL9-mixture A, B or C respectively, pTN pack, pVSV-G) to form an un-linked virus library. Fresh HEK293T cells (13×10^6^) were transduced at low MOI (0.1). The infected Env-genotype-phenotype “linked” cells were applied to the above-described FACS-sorting procedure 48 h after infection. The envelope genes were amplified from the genomic DNA of the collected cells, cloned into pQL9 and analyzed by qPCR. Fresh cells were transfected with the new pQL9 Env/V3-mixture (together with pTN pack and pVSV-G) to produce new virus for low MOI transduction of fresh cells and to enter another cycle of selection. The mean values of two independent experiments are shown. Statistics were calculated using the 1way-ANOVA followed by "Tukey's Multiple Comparison” test (* P<0.05; ** P<0.01; *** P<0.001). The relative amounts per variant of the input mixture, 1^st^ and 2^nd^ cycle are shown as analyzed by qPCR (Efficiency∧^-Ct^ as % of each variant; triplicates) [69]–[71]. A reference primer pair that amplifies a sequence independently of the Env gene inserted was used to determine the total amount of pQL9 Env plasmids in each qPCR, respectively (data not shown).

In order to proceed from a defined mixture of separately transduced cells to a more relevant setting of bulk infected cells, different ratios of pQL9 based chimeric Env/V3-MN and -SF33 plasmids were first mixed and then used to produce corresponding mixed virus batches. These batches were used to infect HEK293T cells at low MOI of 0.1, respectively, thus representing two member “minimal libraries” with different ratios of Env/V3-variant MN (binding) and SF33 (non-binding) (Material and Methods). Each of the subsequent panning cycle consists of six steps (see Figure 1, Step 1–6), followed by quantitative enrichment analyses after every cycle. The Env/V3-variant distributions were analyzed using samples directly before each FACS-sorting (Figure 1, Step 4/Figure 6, Input) and after the 1^st^ and 2^nd^ complete cycle of panning (Figure 1, Step 5/Figure 6, 1^st^/2^nd^). The ratios of the two chimeric Env/V3-variants were calculated based on qPCR analysis (Figure 6 A-C) as well as DNA sequencing of cloned envelope variants (Figure S4).

The results of these first panning experiments demonstrated the ability of the FACS-panning and gating procedure to enrich a “binding” Env (MN) and deplete a “non-binding” Env (SF33). An enrichment rate of ∼3-fold for low Input values of MN (5% MN, 25% MN) (Figure 6 A, B) and an enrichment rate of ∼10-fold for high Input values of MN (50% MN) was achieved after 2 cycles of selection (Figure 6 C; Table 1).

**Table 1:** Enrichment of Env/V3-variant MN.^a^

The specific enrichment of the Env/V3-variant MN is calculated for each FACS-panning.			
Panning	Ratio^a^ of Env recovered after 2 cycles (%)/Env Input (%)		Fold enrichment^b^
	MN	Non-MN	
**pQL9**			
Library Screen (Figure 7 B)	3.09	0.51	6.01
**pQL11**			
Library Screen (Figure 8 B)	4.68	0.23	20.51

a Values obtained by qPCR.

b Calculations were made according to the formula 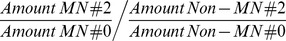 [72].

# cycle of panning.

### FACS-panning: Screen of complete chimeric Env/V3 model-library

**Figure 7:**
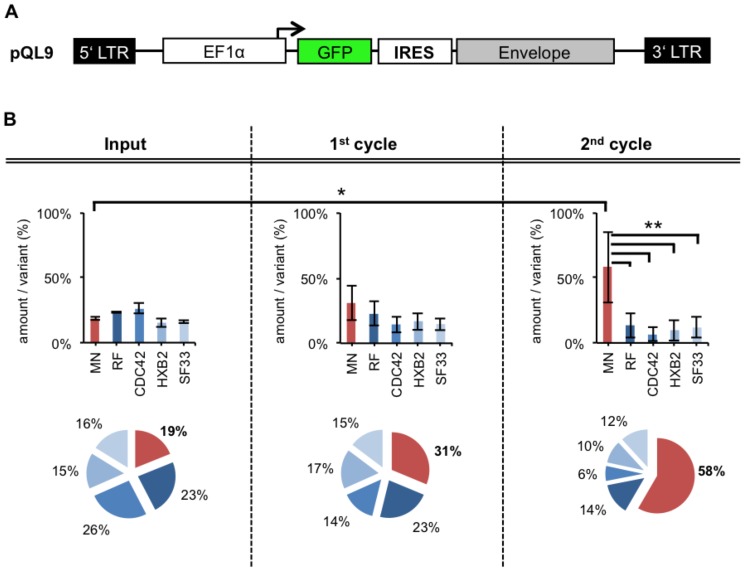
FACS-panning with pQL9 based Env/V3- model library. **A** Schematic overview of pQL9 plasmid **B** HEK293T cells were transduced at low MOI (0,1) of “linked” pQL9 derived chimeric Env/V3-model-library viruses and subjected to a FACS panning and gating procedure 48 h after transduction (as described in Material and Methods and Figure 1/5). Env genes were amplified from genomic DNA of collected cells, cloned into fresh pQL9 and were analyzed by qPCR. New virus batches were produced with the resulting new pQL9 Env-library mixtures for infections of fresh cells, thus entering another cycle of selection. The mean values of four independent experiments are shown. Statistics were calculated using the 1-way-ANOVA- (testing, whether mean values differ) and “Dunnett's” post-test (testing, which mean values differ: * P<0.05; ** P<0.01; *** P<0.001). The relative amounts per variant of the input mixture, 1^st^ and 2^nd^ cycle are shown as analyzed by qPCR (Efficiency∧^-Ct^ as % of each variant). The total amount of pQL9 Env/V3-plasmids in every sample was tested by a reference primer pair that amplifies a sequence independent of the Env genes, respectively (data not shown).

The complete chimeric Env/V3 model-library was screened using the same FACS-panning and gating procedure as described for the evaluation screen above. Briefly, a batch of viruses was produced using the complete chimeric Env/V3 model-library with a plasmid mixture bearing 20% of each V3 variant. Subsequently, fresh HEK293T cells were transduced with low MOI (0.1) and a FACS-panning (Material and Methods) was performed (Figure 7 B).

Equal input values for each chimeric Env variant (∼20%) has been achieved (Figure 7 B; Input). As analyzed by qPCR a continuously increase of the Env/V3-variant MN (19%, 31%, 58%) was achieved by FACS-panning and gating. Statistical analysis of the panning revealed that the Env/V3-variant MN was significantly enriched after the 2^nd^ cycle of panning (Figure 7 B). Selection of the highest-affinity binding chimeric Env/V3-variant-MN out of an equally proportioned Env/V3 model-library mixture was demonstrated.

### FACS-panning: Improved screening efficacy

**Figure 8:**
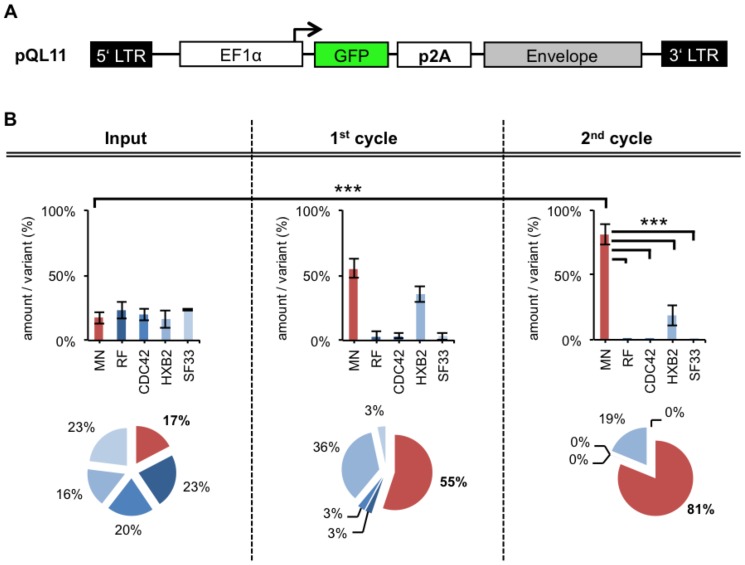
FACS-panning with improved pQL11 based Env/V3 model-library. **A** Schematic overview of the new pQL11 plasmid **B** HEK293T cells were infected at low MOI (0,1) of “linked” pQL11 based chimeric Env/V3-model-library viruses and were applied to a FACS-panning procedure 48 h after infection (as described in Material and Methods and Figure 1/5). Env genes were amplified from genomic DNA of collected cells, cloned into fresh pQL11 and were analyzed by qPCR. New virus batches were produced with the resulting new pQL11 Env-library mixtures for infections of fresh cells, thus entering another cycle of selection. The mean values of three independent experiments are shown. Statistics were calculated using the 1-way-ANOVA- (testing, whether mean values differ) and “Dunnett's” post-test (testing, which mean values differ: * P<0.05; ** P<0.01; *** P<0.001). The relative amounts per variant of the input mixture, 1^st^ and 2^nd^ cycle are shown as analyzed by qPCR (Efficiency∧^-Ct^ as % of each variant). The total amounts of pQL11 Env/V3-plasmids in every sample was tested by a reference primer pair that amplifies a sequence independent of the Env genes, respectively (data not shown).

In order to further increase the enrichment efficacy a revised plasmid, pQL11, was designed to improve the level of Env expression (Figure S5). This was achieved by substituting the IRES with the TaVp2A peptide [17], [42] (Figure 8 A). Hence, the chimeric Env/V3 model library described above was subcloned into the revised pQL11 plasmid.

Consequently, the complete pQL11 based Env/V3 model-library was screened using the same FACS-panning and gating procedure as described in the previous sections. Briefly, new batches of viruses were produced using the complete chimeric Env/V3 model-library with plasmid mixtures bearing 20% of each chimeric V3 variant, respectively. Subsequently, fresh HEK293T cells were infected at low MOI (0.1) and a FACS-panning (Material and Methods) was performed. The distribution of Env/V3-variants during two cycles of selection was monitored by qPCR (Figure 8 B).

Equal Input values for each of the five chimeric Env/V3 variants (∼20%) were achieved (Figure 8 B; Input). In comparison to panning using pQL9 (Figure 7) a more efficient enrichment of the Env/V3-variant MN (Input: 17% MN; 1^st^ cycle: 55% MN; 2^nd^ cycle 81% MN) was achieved by using pQL11. Statistical analysis of the panning revealed that the Env/V3-variant MN was highly significantly enriched after the 2^nd^ cycle of panning (P<0.001). Thus, a more stringent selection of the highest-affinity binding chimeric Env/V3-variant-MN out of an equally proportioned Env/V3 model-library mixture was demonstrated.

The enrichment factors of all FACS-panning experiments in relation to Input amounts are summarized in Table 1.

Whereas the utilization of pQL9 (Env-GFP coupling by IRES) resulted in a ∼6-fold enrichment of the Env/V3 variant MN, the vector improvement leading to pQL11 (Env-GFP coupling by TaVp2A) further improved the enrichment up to ∼20-fold.

Taken together, these proof-of-concept experiments successfully demonstrated the capability to specifically enrich the envelope variant harboring the V3 MN domain, which has been shown to bind with the highest affinity towards 447-52D nMAb, from a pool of competing envelope V3 chimeras.

## Discussion

Recent progress in the field of envelope specific B-cell isolation and monoclonal antibody generation out of chronically infected patients lead to a broad panel of available bnMAbs [11], [18], [43]. As these antibodies can suppress established HIV-1 infection and can protect from SHIV-1 challenge in animal models [19], [44], [45] they are valuable tools for envelope based vaccine design. Several groups pursue a targeted design of envelope immunogens based on available structural information of the bnMAbs in complex with the envelope protein especially the CD4 binding site [20], [46], [47]. Complementary, methods based on protein display and selection could provide additional information of bnMAbs/envelope interactions for vaccine design. Ideally, the method should mimic the natural situation of an envelope protein regarding glycosylation, oligomerisation and surface presentation to allow proper presentation of quaternary epitopes. Various available display systems allow the screening of diverse proteins or peptides out of comprehensive libraries to identify high affinity antigen-antibody interactions, but most of them are not compatible with the requirements [17], [21]–[25], [48]–[52]. The FACS-panning technology developed by our group is based on the display of envelopes on the HEK293T mammalian cell line, which is widely used e.g. as a producer cell-line for functional Env pseudoviruses for HIV-1 neutralization assays [11], [15], [26]–[28], [53], [54]. It allows mammalian glycosylations, as well as a functional Env expression in membrane context like in standardized neutralization assays. Therefore, it should provide access to all of the proposed requirements of envelope presentation, even to the promising quaternary structures of Env [55] as mentioned above.

For the generation of cells expressing envelope variants we chose a lentiviral vector system which allows efficient transduction of various cell types and a stable transgene expression by integration [56]. In contrast to transient transfections [37] were multiple library variants are transfected into a single cell, a low MOI transduction should promote a strong linkage between phenotype and genotype [63], [64], [65]. To further improve our panning strategy the vector was modified to allow a gfp based normalization for differences in expression of the transgenes (Fig.3). As other mammalian cell displays select directly for high-level expression [63], [64], [65], we adjusted our FACS sorting procedure to select for most efficient antibody binding relative to gfp normalization (Fig.5). By the use of a more stringent coupling of transgene and gfp on a translational level we could further improve this selection method (Fig. S5).

As a proof-of-concept tool, a chimeric, 5-member Env/V3 model-library was created based on knowledge of earlier experiments by Gorny et al. [34]. They determined the dissociation constants (K_D_) of V3-peptides from different HIV-1 strains to the biochemically well-characterized nMAb 447-52D [32]–[34]. While the generation of Env/V3-variants with diverging affinity to 447-52D could be achieved using complete cell-surface exposed envelopes, a different order of affinities between the V3 sequences with regard to 447-52D binding was observed, underlining the importance of binding studies in the context of a full length extracellular envelope.

Our FACS-panning and gating procedure could successfully discriminate between the five chimeric envelope variants as detected by two different methods: Sanger sequencing of selected clones is an established and widely used method for the determination of selected variants in display and selection methods [53], [65]. It represents however only a drawn sample out of all selected variants and their significance is strongly dependent on the size of variability in the input material. Therefore we developed a qPCR enrichment-analysis based on total genomic DNA isolated out of cells from the high binding population. As the number of variants analyzed in this proof of concept study was low, both methods detected the enrichment rate equally (Fig. S6).

By the use of the pQL11 vector we could detect an enrichment rate of 20-fold after two rounds of selection. Other display technologies reported higher enrichment rates, which were however calculated only based on the selection between binders versus non-binders using a mixture of cell populations [37], [53]. Hence, the FACS-panning demonstrated herein, offers a unique solution for bnMAb-dependent, affinity-enrichment of Env proteins expressed on mammalian cell surfaces following transduction with a swarm of lentiviral particles.

The identification and use of complete trimeric envelopes in an active immunization scenario is considered very promising regarding the elicitation of broadly neutralizing antibodies in vaccinees [57]. It remains to be demonstrated in future studies to which extent trimeric envelopes that were selected based on improved antigenicity in terms of in vitro binding properties to one or several of the available bnMAbs will be able to elicit a polyclonal antibody response in vivo exhibiting a broadened neutralization profile. As a necessary prerequisite, future screening efforts analyzing more comprehensive Env libraries have to demonstrate, to which extent it will be possible to isolate specific Env conformations using e.g. potent bnMAb (e.g. VRC01 [58]; PG9/16 or PGT123 [11]). Additionally, it might be particularly interesting to isolate a series of related Env vaccine candidates, which bind to germline B-cell receptor (BCR), calculated transition stages and the corresponding mature bnMAbs. The consecutive vaccination with the accordingly selected envelope variants might contribute towards guiding the formation of germinal centers and eliciting broadly neutralizing antibody responses [59]–[61].

In conclusion, we have herein provided proof-of-principle that the mammalian cell-based display of complete HIV envelopes in conjunction with a FACS procedure utilizing bnMAbs for panning is capable of selecting high affinity binders from Env libraries. Further studies will have to be directed towards further challenging and possibly improving the described technology in order to enable panning of high affinity binders from more comprehensive envelope libraries. These will comprise at least several hundreds of envelope variants from either genetically engineered envelopes or libraries generated from PBMCs of patients or vaccinees showing a certain breadth of neutralization. Future studies will have to demonstrate to what extent envelopes binding with increased affinity to the available bnMAbs and/or their germline versions or intermediate precursors are capable of inducing antibody responses with the desired breadth.

## Materials and Methods

### Construction of pQL9 and pQL11

The lentiviral plasmid pWPXLd (Addgene plasmid #12258) [62] was used as a starting construct. Because of the presence of Esp3I sites, the original backbone of pWPXLd was substituted by non- Esp3I containing plasmid backbone part of pcDNA3.1(+) in sequentially steps: (I) PCR amplification of the MCS (multiple cloning site) of pcDNA3.1(+) and blunt-end ligation with pPCR-Script-Amp linearized with EcoRV. (II) Subsequently, the plasmid was linearized with EcoRV/XbaI within the MCS and ligated with the lentiviral part of pWPXLd obtained by restriction with SspI/XbaI (5.7 kb fragment). (III) The substitution of eGFP within the WPXLd part by a CcdB (killer-gene [63]) expression cassette flanked by two Esp3I restriction sites, cloned via MluI/NdeI. Further on these plasmids were propagated in DB3.1 bacteria (CcdB resistant). (IV) To further decrease the plasmid size, another substitution of the plasmid backbone was performed by ligation of the lentiviral part (EcoRI; blunt-ended; 5.4 kb) to a minimal part of the pcDNA3.1(+) plasmid backbone (PciI/MfeI; blunt-ended; dephosphorylated; 1.9 kb). (V) A GFP gene (GenBank # U55763) was cloned into the MCS of pMACS LNGFR-IRES (#130-091-887, Miltenyi Biotech) via EcoRI/BamHI. Subsequently, an expression cassette consisting of GFP, synthetic Intron [64] and an IRES site (internal ribosome entry site) (pQL9) or GFP, TaVp2A [42] (pQL11) were introduced upstream of the CcdB cloning site (via PCR amplification, extending the Insert with MluI/NheI), resulting in the final pQL9 and pQL11 plasmids respectively.

### The QL cloning procedure

For cloning of DNA Inserts (envelopes) into pQL9 or pQL11 a combined restriction and ligation process was used. The reaction is divided into two steps. (I) 2 µL 10 x Tango Buffer (Fermentas), 2 µL 10 mM DTT, 100 ng pQL9/11, 100 ng Insert, 1 µL Esp3I (Fermentas) and addition of H_2_0 to reach 20 µL. This restriction mixture containing the pQL plasmid vector and the DNA fragment to be inserted (Insert) was incubated in a PCR cycler for 45 min at 37°C. Meanwhile a second reaction for the ligation was prepared. (II) 3 µL 10 mM ATP, 1 µL 10 x Tango Buffer, 1 µL 10 mM DTT, 1 µL T4-Ligase (NEB) addition of H_2_0 to reach 10 µL. Subsequently, the second was added to the first reaction-mixture. The combined reactions were incubated in a PCR cycler at 25°C, 37°C, 25°C for 45 min each. Finally, an incubation step at 65°C for 10 min inactivates the enzymes in the reaction mixture. A subsequent selection for positively cloned plasmids is done by transformation of the CcdB sensitive bacterial strain DH10B.

### Construction of the chimeric Env/V3 model-library

A non-binding envelope of HIV-1 isolate 96ZM651 (Accession number: AF286224) [65] was chosen as backbone for the substitution of its complete V3 region with those of different 447-52D antibody binding HIV-1 isolates [34].

The DNA sequences comprising the complete V3 regions of different HIV-1 isolates were adapted to human codon usage. The plasmid pcDNA3.1-96ZMgp145 was used as template consisting of a human codon adapted gp145 envelope coding region of 96ZM651 cloned into the MCS of pcDNA3.1(+). The V3 region of the envelope 96ZM651 was substituted by fusion PCR, inserting V3 regions of different isolates (see Table S1). Finally five different 96ZM651 based Env/V3-variants were chosen to form an Env/V3 model-library of chimeric Envs that only differ in the small region of V3. The constructs were cloned into the lentiviral pQL9 and pQL11 plasmid system by the QL cloning procedure. The plasmids were validated by restriction and sequence analysis (data not shown).

### Validating process for un-biased amplifications of the chimeric Env/V3 model-library

Although all pQL Env/V3-plasmids share the same plasmid and Env background, a bias in the selection of variants remained possible. Such influences could bias the validity of results obtained by the designed Env/V3 model-library. Especially, the influence of a respective pQL Env/V3-library plasmid on bacterial growth after transformation could lead to an unspecific bias in the enrichment procedure and was therefore carefully analyzed. The bacterial growth-rates and plasmid yields of separate over-night cultures were analyzed by OD_600_ measurement, respectively. The bacterial growth-rates were measured in five replicates. All strains consistently had doubling-times of 78±4 min. Subsequently, isolated plasmid DNA were tested for correctly sized bands after enzymatic restriction (AleI). All samples consistently showed correctly sized restriction patterns, as well as equal plasmid DNA yields (data not shown). Thus, a bias resulting from varying bacterial plasmid propagation of the different Env/V3-variants was excluded.

Furthermore, the accuracy of enrichment measurements is dependent and therefore limited by the sample size analyzed. Preliminary measurements were based on sequencing random samples of single bacterial clones (Figure S4, S6), resulting in enrichment rates accurate ±10% within a confidence level of 95% related to the parent population [66].

Further improvement in accuracy was achieved by establishing a qPCR protocol allowing the specific detection of each Env/V3-variant within a mixture of DNA (Figure S1), resulting in an increase of statistical accuracy within a confidence level of 95%, up to ±0.05% [66].

Additionally, efficiencies of all primer pairs designed for qPCR purposes as well as for the primer pairs that were used to amplify complete Env genes were determined. Lastly, an equal efficiency of all primer pairs was observed (Figure S1). Taken together, no bias was detected that could have shifted the enrichment results additionally to the FACS-sorting procedure.

### Transfection of HEK293T cells

Transfection of HEK293T cells was done according to common transfection protocols [67]. Briefly, HEK293T cells were seeded to reach 80% confluence on the day of transfection. As an example, 5×10^5^ cells were seeded in 2 mL DMEM (#41966, Invitrogen) supplemented with 10% FCS (10270-106, GIBCO), 1% Pen/Strep (P06-07100, PAN Biotech) medium into a 6-well plate the day before transfection. Directly before transfection, the medium was replaced by 1 mL of DMEM without supplements and incubated as before until transfection. A transfection mixture was prepared by diluting 2 µg of DNA into 100 µL DMEM without supplements, adding 8 µL PEI (Polyethylenimine in sterile water at 1 mg/mL) and incubation of 10 min at RT after vigorously mixing on a vortex instrument. The prepared mixture was pipetted to the cells by lifting the plate on one side and releasing the mixture directly below the surface of the medium, mixed by swirling and incubated for 6 h before the medium was replaced by 2 mL DMEM. After 24 to 72 h the cells or the supernatant were harvested. If smaller or greater vessels were used, the amount of cells, DNA, PEI and the amount of medium were scaled in relation to the surface of the vessel.

### Production of virus batches

Transfection of a three-component plasmid mixture of pTN-pack/pQL9 or pQL11 based plasmids/pVSV-G (see Table S2) into HEK293T cells initiated the production of viruses. The plasmids were mixed in the ratio 4∶3∶1, with the total amount of 30 µg DNA. HEK293T cells were transfected in a 15 cm dish according to the previously described protocol for the transfection of HEK293T cells. After 72 h of incubation the supernatant was cleared by centrifugation at 4°C, 3000×g for 15 min, aliquots were stored at −80°C or transferred onto a 30% sucrose cushion by pipetting the sucrose solution carefully below the supernatant. After ultracentrifugation (BD, Optima L90K) at 4°C, 100000×g for 2 h the pellet was re-suspended in 300 µL of DMEM medium over night at 4°C and stored in 50 µL portions at −80°C.

### Infection and determination of multiplicity of infection (MOI)

The day before infection 13×10^6^ cells in 30 mL DMEM (10% FCS, 1% Pen/Strep) were seeded into a 15 cm dish, or 2.5×10^4^ cells in 0.5 mL into each well of a 6-well plate. Directly before infection the medium was replaced by freshly prepared 20 mL, or 0.5 mL, DMEM containing 10 µg/mL Polybrene (10 µg/µL Hexadimethrine bromide in sterile water, #H9268, Sigma). Virus was thawed slowly on ice or was used directly after production. Various amounts of virus were pipetted to the cells depending on the experiment by lifting the dish or plate on one side and releasing the virus directly below the surface of the medium, mixed by swirling and incubated for 24 h before the medium was replaced by 30 mL or 0.5 mL fresh DMEM medium. The cells were assayed 48 h after infection. In order to determine the amounts needed to achieve low MOI (≤0.2) infections, the infectivity of each virus production was deduced by infecting cells with different amounts of virus. GFP positive cells were counted after 48 h post infection by FACS analysis and related to the volume of virus applied. The MOI was estimated to be in a range of highest probability for singly infected cells (low MOI: ≤0.2) by correlating the measured infection rate (% positive cells) and the expected MOI according to the Poisson distribution (Figure S2). Briefly, at MOI 0.2 the probability (P(n)) that a cell is infected by 0 (n) virus is 81.87% (the negative cells). All other cells are infected by 1 (n) virus 16.37%, 2 (n) viruses 1.64% and so on. Therefore, most cells getting infected at MOI ≤0.2 are singly infected cells.

### FACS-analysis

Cells were detached with PBE (PBS +0.5% FCS, 2 mM EDTA, 1 mg/mL NaN_3_) and centrifuged at 200×g for 5 min at 4°C. Subsequently, cells were incubated with 0.001–10 µg/mL 447-52D antibody (AB014, Polymun) diluted in PBE at 4°C. Adjacent incubation, cells were washed 3 x with 1 mL ice cold PBE, centrifuged at 200×g for 5 min at 4°C. A secondary antibody anti-human-IgG-APC (#109-136-098, Jackson) diluted in PBE was applied for 1 h at 4°C. Another 3 x with 1 mL ice cold PBE washing step followed and cells were centrifuged at 200×g for 5 min at 4°C. Finally, cells were suspended in PBE and subjected to cytometric analysis. Un-transfected cells were treated equally and used as background control. Env coupled GFP expression signals (MFI) were used to calculate normalized Env expression signals (MFI) when the affinity of 447-52D antibody was determined or were used for gating desired cell populations in the FACS-panning procedure. Cell preparations for cytometric analysis were probed, using FACS Canto II or FACS Aria (Becton Dickinson).

### FACS-panning: Affinity enrichment and isolation of Env/V3-variants

Cells were prepared according to the protocol described in the previous section and were finally re-suspended in 1 mL PBE per one 15 cm dish and filtered with a 30 µm pre-separation filter (#130-041-407, Miltenyi Biotech). Cells were subjected to cytometric sorting utilizing a FACS Aria instrument. The instrument was set to “single cell mode” to obtain the most accurate counts for the sorting procedure. In this mode drops containing two target events are discarded. When a particle is detected matching the criteria for separation, an electrical charge is applied just as the droplet containing this particle breaks off from the liquid stream. As the charged droplet passes strongly charged deflection plates, it can be separated and collected [68]. The genomic DNA of the sorted cells and a portion of cells before sorting were prepared according to the manufacturer's instructions (#51104, Qiagen) but without addition of carrier DNA and limited to 20 µL 10 mM Tris-elution buffer for increasing the DNA concentration. To amplify viral integrated envelope genes the genomic DNA was used as a template for Nested-PCR reactions (Primer, see Table S1).

### Quantitative PCR (qPCR)

In order to determine the efficiency of PCR reactions, a qPCR standard curve analysis utilizing a StepOnePlus (Applied Biosystems) device was performed. Genomic or plasmid sample DNA was prepared and a 5-fold dilution series was analyzed according to the manufacturer's instructions (Applied Biosystems, as well as Finnzymes). To determine the amount of different envelope coding genes in a mixture of DNA, a qPCR reaction containing one specific reverse-primer was designed for each gene analyzed. Consequently, the probe and forward-primer was designed to bind at a region with equal sequences for all different Env/V3-variants. Furthermore, one additionally reverse-primer was designed that binds at a region with equal sequences for all variants and therefore was used as a reference primer, determining the total amounts of the applied variants (Table S1, Figure S1). The amount of the sample DNA used varies according to the experimental setting. For both, SYBR Green chemistry and probe-based chemistry, the manufacture's reaction protocols were used (DyNAmo F-415, F-455, Finnzymes).

## Supporting Information

Figure S1 **Verification of qPCR analyses.** **A** Schematic overview of qPCR components used. Consensus sequences are color-coded. Sequences corresponding to Env are shown in black and those for pQL plasmids are depicted in grey. Oligonucleotides like primers or probes are shown as grey, black or colored arrows. The colors and orientation visually connect the plasmid sequence to its oligonucleotide binding area. The Probe is 5′ labeled with FAM and 3′ quenched by BHQ1. **B** PCR efficiencies for all primer pairs used on the V3 plasmids were calculated in order to demonstrate comparable efficiencies between the different V3 constructs. Efficiencies for primer 8H1 and 8H2 were tested with SYBR Green (mean and SD of two separate experiments). The other primers were tested with probe-based qPCR. **C** Probe based qPCR amplifications on plasmid mixtures were done to verify the specificity of every possible combination of the Env V3-variants used. The name of the colored samples indicates whether the tested variant is present or absent. Triplicates of 5 µL of every sample mixture (1 ng/µL) were tested with its corresponding specific Primer-r, but with equal Primer-f and Probe. Rn values (normalized Reporter) are the ratio of the fluorescence emission intensity of the reporter (Probe) to the fluorescence emission intensity of the passive reference dye (i.e. ROX). Rn is plotted against the PCR cycle number to illustrate the amplification of PCR products.
(TIF)

Figure S2 **The Poisson distribution is used to calculate the probability for cells to get infected with a specific number of viruses at a given MOI.** At low MOI (0.1–0.5) the average fraction of cells that will become infected (P_(n>0)_ = 1– P_(n = 0)_) is approximately equal to the MOI (m). Furthermore, low MOI infections lead predominantly to non- or single infection events. This is especially true for the applied MOI of 0.1 (green), but also remains true until the MOI of 0.5, as illustrated.
(TIF)

Figure S3 **Ratio of 447-52D (APC) and GFP signals for infected cells.** HEK293T cells (3×10^5^) were infected with pQL9 Env V3-MN virus with the MOI indicated (MOI: 0.05–0.5) and stained with 50 µL 447-52D antibody (10 µg/mL) 48 h after infection. FACS analysis is shown for the different MOI of the applied virus as the MFI of gated living cells. The ratio of 447-52D/GFP is shown with a secondary axis to indicate the connection between the expression levels of the envelope variant MN and GFP. The mean values of two independent experiments are shown.
(TIF)

Figure S4 **FACS-panning using different ratios of Env V3-variants MN and SF33.** The Panning procedure was performed as described in Figure 6 A-C. Additionally to the performed qPCR analysis (Figure 6) the relative amounts per variant of the input-mixture, 1^st^ and 2^nd^ round were analyzed by sequencing one 96-well plate of single clones each. The mean values of two independent panning experiments are shown. Statistics were calculated using the 1way-ANOVA followed by "Tukey's Multiple Comparison” test (* P<0.05; ** P<0.01; *** P<0.001).
(TIF)

Figure S5 **Improved linkage of coexpression.** Representative samples of **A** pQL9-MN and **B** pQL11-MN low MOI infected HEK293T cells were analyzed. A scatter plot of all 447-52D antibody and GFP positive cells is shown. Linkage of coexpression were further analyzed by calculating a linear regression curve (purple) and R^2^ values respectively. The higher R^2^ value depicted in **B** indicates a stronger linear approximation for pQL11 based coexpression of envelope and GFP, than for pQL9.
(TIF)

Figure S6 **FACS-panning by sequencing single clones.** The Panning procedure was performed as described for Figure 7. Additionally to the performed qPCR analysis the distribution of variants were analyzed by sequencing one 96-well plate of single clones for Input samples and after each cycle. The mean values of four independent experiments are shown. Statistics were calculated using the 1-way-ANOVA- (testing, whether mean values differ) and “Dunnett's” post-test (testing, which mean values differ: * P<0.05; ** P<0.01; *** P<0.001).
(TIF)

Table S1 Oligonucleotides. A complete list of all oligonucleotides that were used for this project.
(DOC)

Table S2 Plasmid constructs. A complete list of all plasmids that were used for this project.
(DOC)
