# Supplementary figures and images for: A Mammalian Cell Based FACS-Panning Platform for the Selection of HIV-1 Envelopes for Vaccine Development
Source: PLoS One. 2014 Oct 3;9(10):e109196. doi: 10.1371/journal.pone.0109196 (PMC4184847; doi:10.1371/journal.pone.0109196)

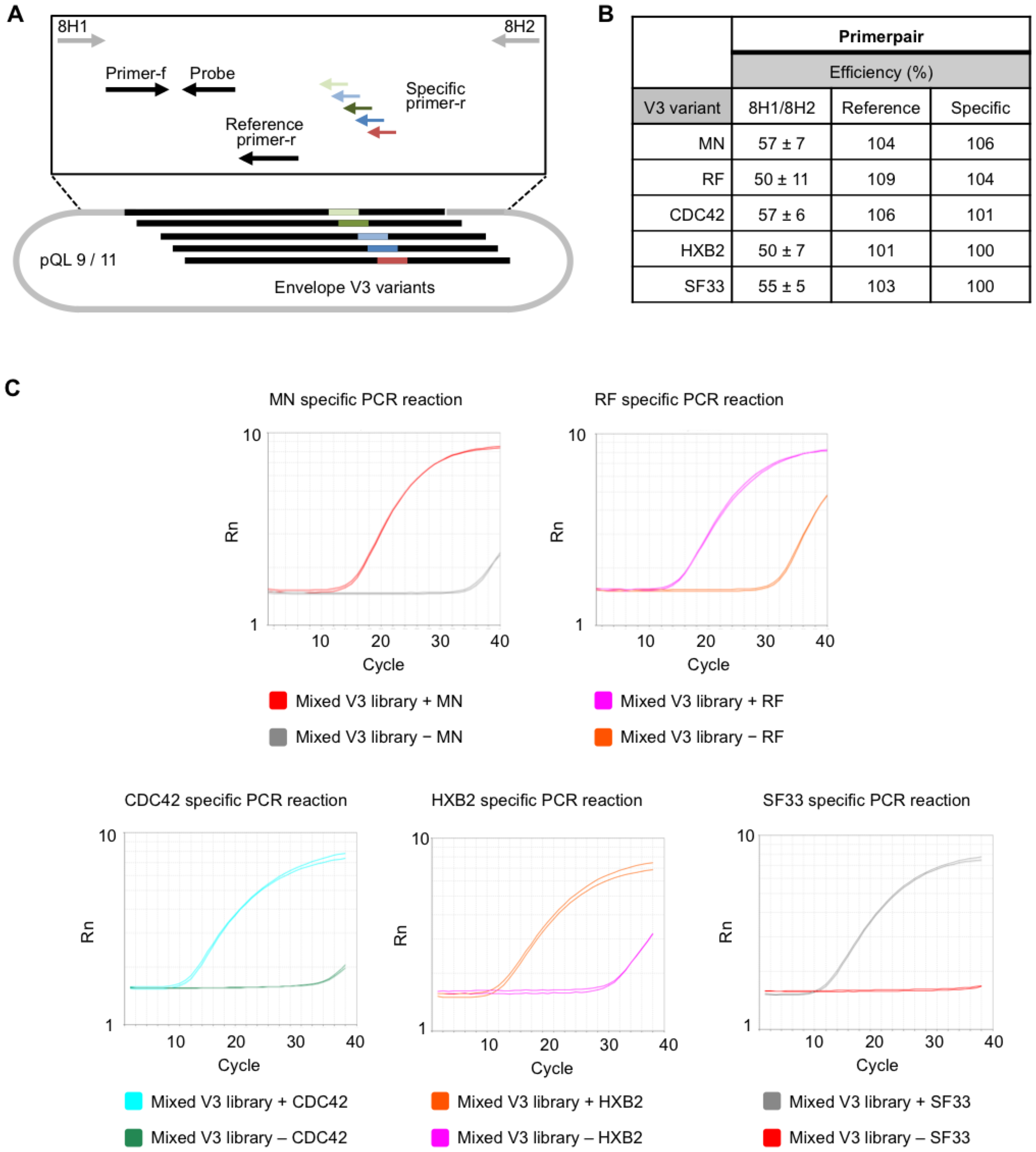

Supplement: Figure S1 — Verification of qPCR analyses. A Schematic overview of qPCR components used. Consensus sequences are color-coded. Sequences corresponding to Env are shown in black and those for pQL plasmids are depicted in grey. Oligonucleotides like primers or probes are shown as grey, black or colored arrows. The colors and orientation visually connect the plasmid sequence to its oligonucleotide binding area. The Probe is 5′ labeled with FAM and 3′ quenched by BHQ1. B PCR efficiencies for all primer pairs used on the V3 plasmids were calculated in order to demonstrate comparable efficiencies between the different V3 constructs. Efficiencies for primer 8H1 and 8H2 were tested with SYBR Green (mean and SD of two separate experiments). The other primers were tested with probe-based qPCR. C Probe based qPCR amplifications on plasmid mixtures were done to verify the specificity of every possible combination of the Env V3-variants used. The name of the colored samples indicates whether the tested variant is present or absent. Triplicates of 5 µL of every sample mixture (1 ng/µL) were tested with its corresponding specific Primer-r, but with equal Primer-f and Probe. Rn values (normalized Reporter) are the ratio of the fluorescence emission intensity of the reporter (Probe) to the fluorescence emission intensity of the passive reference dye (i.e. ROX). Rn is plotted against the PCR cycle number to illustrate the amplification of PCR products. (TIF) [file pone.0109196.s001.tif]

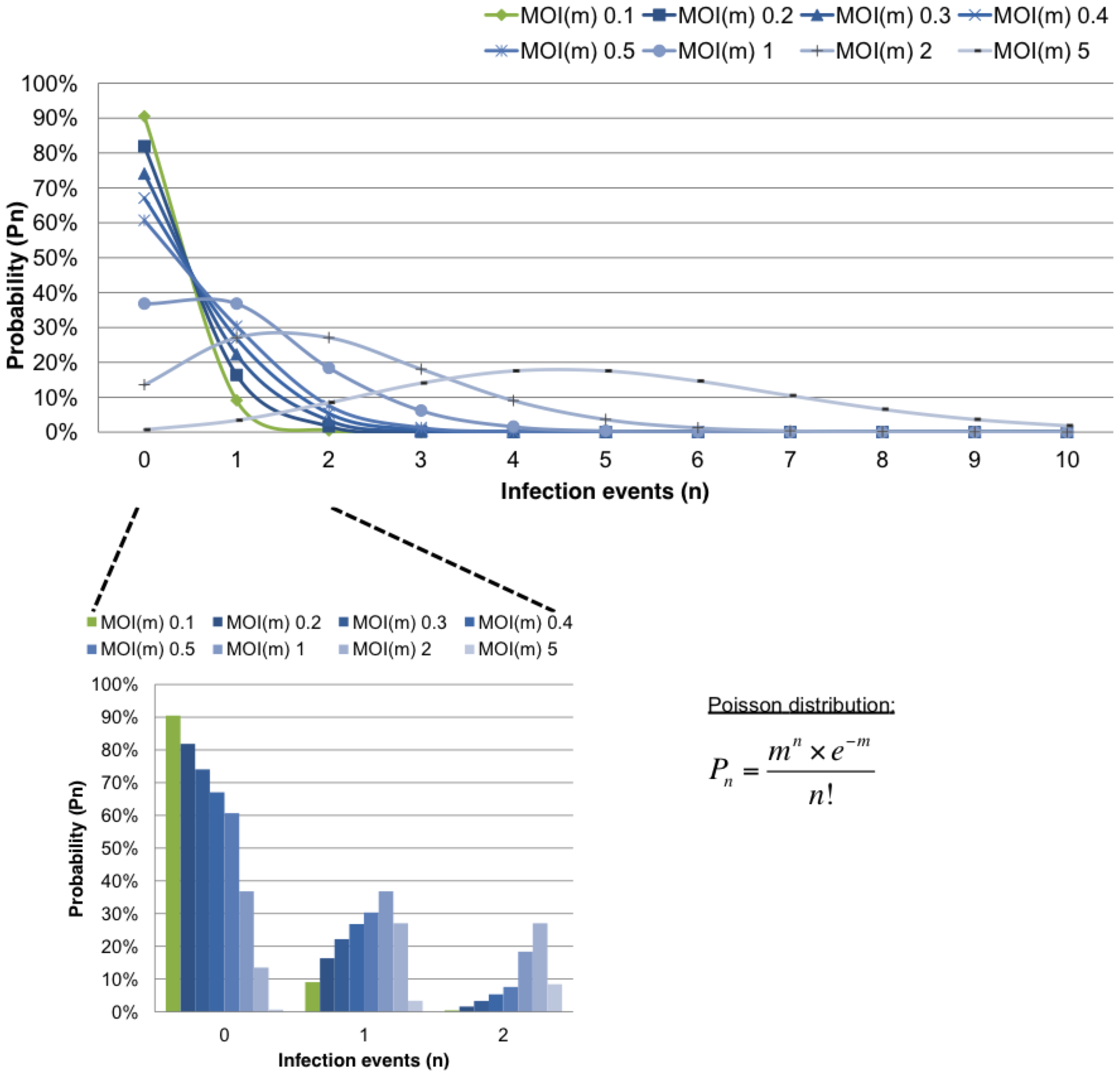

Supplement: Figure S2 — The Poisson distribution is used to calculate the probability for cells to get infected with a specific number of viruses at a given MOI. At low MOI (0.1–0.5) the average fraction of cells that will become infected (P(n>0) = 1– P(n = 0)) is approximately equal to the MOI (m). Furthermore, low MOI infections lead predominantly to non- or single infection events. This is especially true for the applied MOI of 0.1 (green), but also remains true until the MOI of 0.5, as illustrated. (TIF) [file pone.0109196.s002.tif]

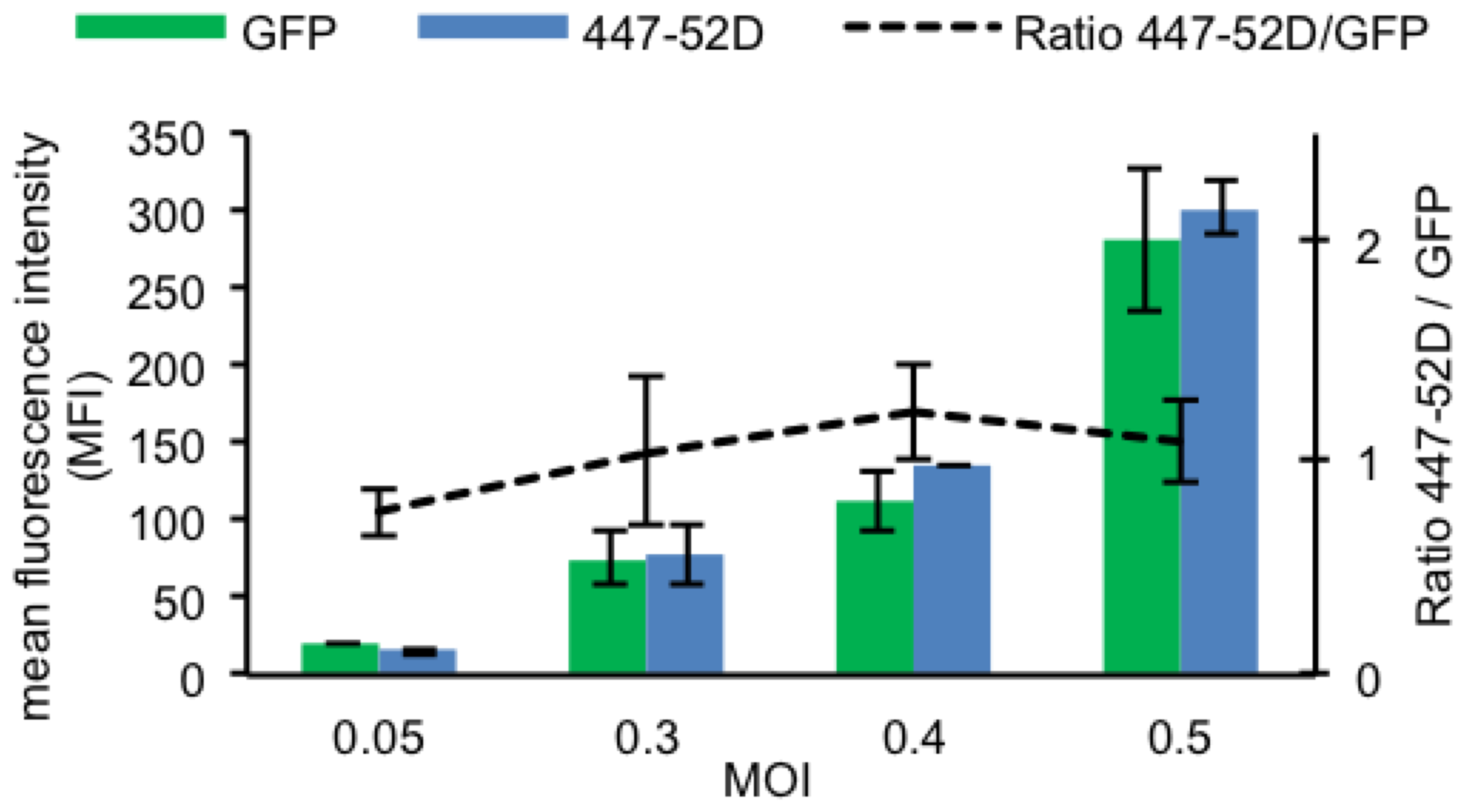

Supplement: Figure S3 — Ratio of 447-52D (APC) and GFP signals for infected cells. HEK293T cells (3×10^5^) were infected with pQL9 Env V3-MN virus with the MOI indicated (MOI: 0.05–0.5) and stained with 50 µL 447-52D antibody (10 µg/mL) 48 h after infection. FACS analysis is shown for the different MOI of the applied virus as the MFI of gated living cells. The ratio of 447-52D/GFP is shown with a secondary axis to indicate the connection between the expression levels of the envelope variant MN and GFP. The mean values of two independent experiments are shown. (TIF) [file pone.0109196.s003.tif]

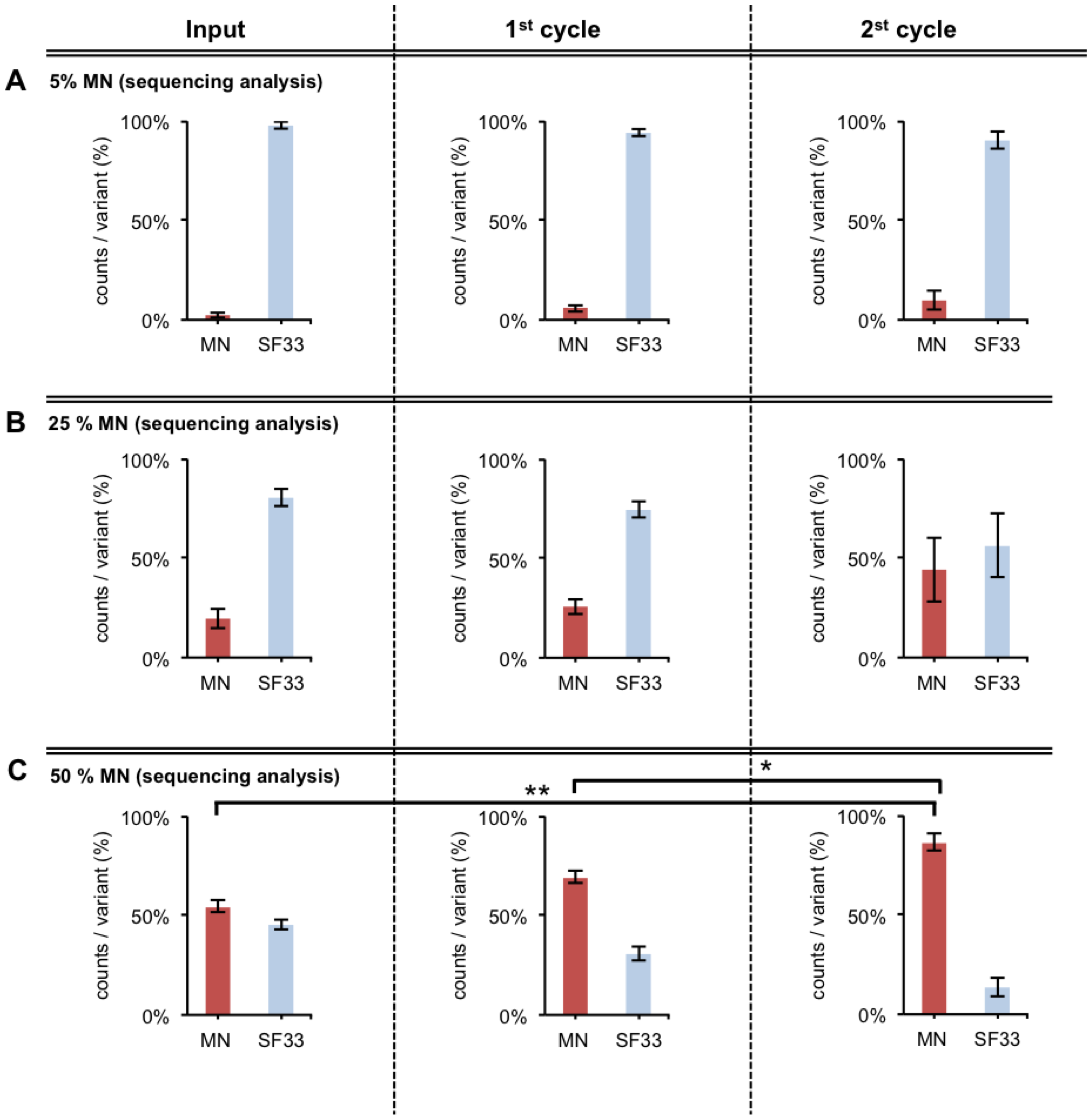

Supplement: Figure S4 — FACS-panning using different ratios of Env V3-variants MN and SF33. The Panning procedure was performed as described in Figure 6 A-C. Additionally to the performed qPCR analysis (Figure 6) the relative amounts per variant of the input-mixture, 1st and 2nd round were analyzed by sequencing one 96-well plate of single clones each. The mean values of two independent panning experiments are shown. Statistics were calculated using the 1way-ANOVA followed by "Tukey's Multiple Comparison” test (* P<0.05; ** P<0.01; *** P<0.001). (TIF) [file pone.0109196.s004.tif]

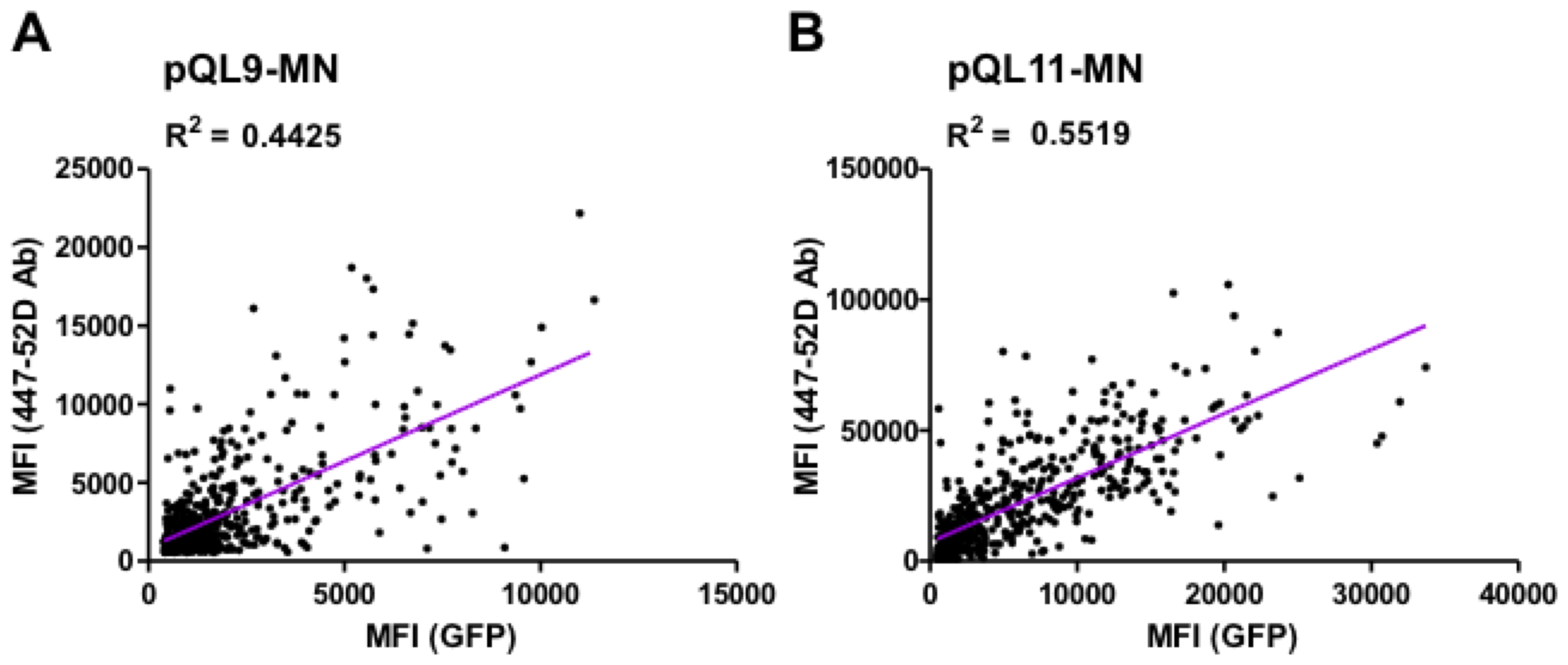

Supplement: Figure S5 — Improved linkage of coexpression. Representative samples of A pQL9-MN and B pQL11-MN low MOI infected HEK293T cells were analyzed. A scatter plot of all 447-52D antibody and GFP positive cells is shown. Linkage of coexpression were further analyzed by calculating a linear regression curve (purple) and R2 values respectively. The higher R2 value depicted in B indicates a stronger linear approximation for pQL11 based coexpression of envelope and GFP, than for pQL9. (TIF) [file pone.0109196.s005.tif]

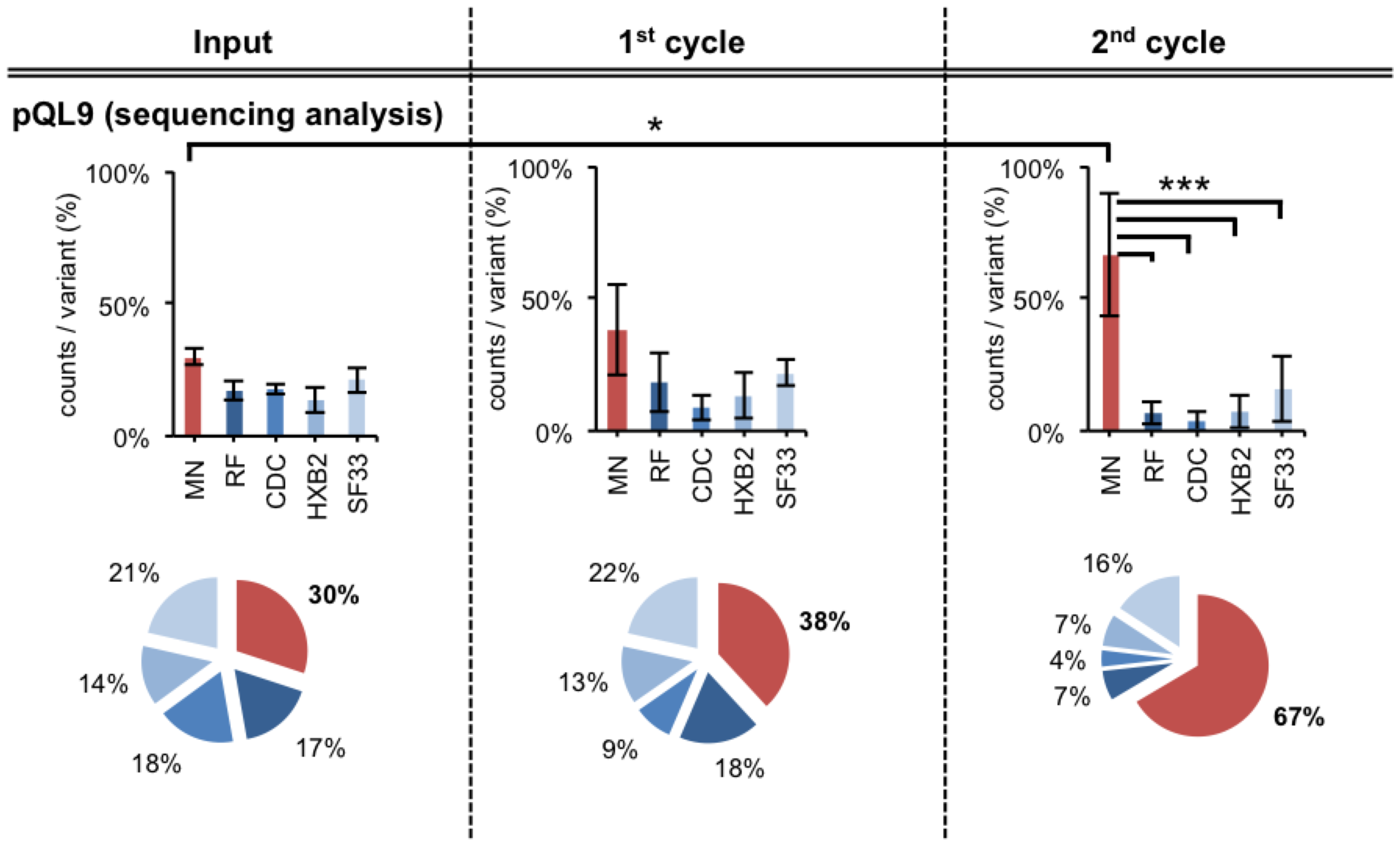

Supplement: Figure S6 — FACS-panning by sequencing single clones. The Panning procedure was performed as described for Figure 7. Additionally to the performed qPCR analysis the distribution of variants were analyzed by sequencing one 96-well plate of single clones for Input samples and after each cycle. The mean values of four independent experiments are shown. Statistics were calculated using the 1-way-ANOVA- (testing, whether mean values differ) and “Dunnett's” post-test (testing, which mean values differ: * P<0.05; ** P<0.01; *** P<0.001). (TIF) [file pone.0109196.s006.tif]
