# Supplementary material for: A Mammalian Cell Based FACS-Panning Platform for the Selection of HIV-1 Envelopes for Vaccine Development
Source: PLoS One. 2014 Oct 3;9(10):e109196. doi: 10.1371/journal.pone.0109196 (PMC4184847; doi:10.1371/journal.pone.0109196)
Supplement: Table S1 — Oligonucleotides. A complete list of all oligonucleotides that were used for this project. (DOC) [file pone.0109196.s007.doc]

**Table S1. Oligonucleotides**

| **ID** | **Name** | **Sequence 5’🡪3’** |
| --- | --- | --- |
|  | **V3 library construction** |  |
| 5F3 | THB-5F3-fwd-QL-ZM96 | ATAATACGTCTCGCTAGCATGGGAGTGCGGGAGATCCTGCGG |
| 5G5 | THB-5G5-rev-QL-ZM96 | ATATTCGTCTCCTCGAGCTAGTAGCCCTGCCGCACTCTGT |
| 4B7 | THB V3-InsertMN fwd | TCGGACCTGGGAGAGCTTTTTACACCACCAAAAACATCATCGGCACCATTAGACAGGCTCATTGTAACATCAGCCGGACCAACTG |
| 4B8 | THB V3-InsertMN rev | TGGTGGTGTAAAAAGCTCTCCCAGGTCCGATGTGAATCCGTTTCCGTTTGTTGTAGTTTGGTCTAGTACACACGATCTCGATGGATCTGTTC |
| 4B9 | THB V3-InsertRF fwd | AAGGACCTGGGAGAGTCATCTACGCTACTGGCCAGATCATTGGCGACATCCGAAAAGCTCACTGTAACATCAGCCGGACCAACTG |
| 4C1 | THB V3-InsertRF rev | CAGTAGCGTAGATGACTCTCCCAGGTCCTTTGGTGATGGATTTCCGGGTATTGTTGTTGGGTCTAGTACACACGATCTCGATGGATCTGTTC |
| 4C2 | THB V3-InsertCDC42 fwd | TGGGACCTGGCCGAGTCTGGTACACAACTGGCGAGATTCTCGGAAACATTCGGCAGGCTCACTGCAACATCAGCCGGACCAACTG |
| 4C3 | THB V3-InsertCDC42 rev | CAGTTGTGTACCAGACTCGGCCAGGTCCCAGAGTGACTCGTTTTCGGGTGTGGTTGTTGGGTCTAGTACACACGATCTCGATGGATCTGTTC |
| 4C6 | THB V3-InsertHXB2 fwd | TTCAGAGAGGACCTGGACGAGCTTTTGTGACAATCGGGAAAATCGGCAACATGAGACAGGCTCACTGTAACATCAGCCGGACCAACTG |
| 4C7 | THB V3-InsertHXB2 rev | TCACAAAAGCTCGTCCAGGTCCTCTCTGAATTCTAATCCGTTTCCGGGTATTGTTGTTGGGTCTAGTACACACGATCTCGATGGATCTGTTC |
| 4C8 | THB V3-InsertSF33 fwd | CTGGACCTGGCAAAGTGCTCTACACTACCGGGGAAATCATTGGAGACATTCGGAAGGCCTACTGTAACATCAGCCGGACCAACTG |
| 4C9 | THB V3-InsertSF33 rev | CGGTAGTGTAGAGCACTTTGCCAGGTCCAGATGTGATCCGTCGCCGTCTATTGTTGTTGGGTCTAGTACACACGATCTCGATGGATCTGTTC |
| 4D9 | ZM-96-seq-For (sequencing) | gacagacgacagcgagacagg |
| 4E1 | ZM96-seq-Rev (sequencing) | CTCTCAGCAGGTTGCTCTGC |
| 4F1 | THB ZM96 seq-1-fwd (sequencing) | ATCCCCATCCACTACTGCG |
|  |  |  |
|  | **QL-plasmid construction** |  |
| 4A1 | T7-prom fwd | taatacgactcactata |
| 1A4 | BGH-Rev | GCAACTAGAAGGCACAGTCGAGG |
| 5G6 | THB-5G6-fwd-QL-Esp3I-ccdB | ATAATAACGCGTGCTAGCGAGACGCCGGAATTGCCAGCTGGGG |
| 5G7 | THB-5G7-rev-QL-Esp3I-ccdB | TATATACATATGCTCGAGGAGACGTTATTAAATGCCCCAAAACATCAGG |
| 4G7 | GFP-FOR-EcoRI | CTCTCTgaattcatggtgagcaagggcg |
| 4G8 | GFP-Rev-XbaI | TCCTGAagatctTTACTTGTACAGCTCGTCC |
| 6H1 | THB-6H1-Nde1-IRES-fwd | TATTcatatgcgagcatgcatctaggg |
| 6H2 | THB-6H2-Nde1-GFP-reverse | TATTcatatgTTACTTGTACAGCTCGTCCATG |
| 6F5 | THB-6F5-GFP-rev | TTACTTGTACAGCTCGTCCATGC |
| 6H3 | THB-6H3-YFP-SV40pA-rev | GCAGTGAAAAAAATGCTTTATTTG |
| 6I7 | THB-6I7 ccDB-PmeI-f | TAATAgtttaaacGCTAGCGAGACGCCGGAATTGCCAGCTGGGG |
| 6I8 | THB-6I8 ccDB-BamHI-r | ATATAggatccCTCGAGGAGACGTTATTAAATGCCCCAAAACATCAGG |
| 6I9 | THB-6I9-f-NdeI-syn.Intron | TATTcatatgctaagtaaggatccactagtaacgg |
| 8B2 | THB-8B2-f-MluI-GFP | TATTacgcgtGCCATGGTGAGCAAGGGCGAGGAGCTGT |
| 8B3 | THB-8B3-r-NheI-IRES | TATTgctagcCGGTCCGCTTTGCGGACTGATGGGGAA |
|  | Sequencing primer |  |
| 8D5 | THB-8D5-QL9-seq | aggacatcatcagcctgtggg |
| 8D6 | THB-8D6-QL9-seq | CTGGAAATCACCACCCACAG |
| 8D7 | THB-8D7-QL9-seq | agcatcaccctgaccgccc |
| 8D8 | THB-8D8-QL9-seq | acagaacgagaaggacctgc |
| 8D9 | THB-8D9-seq-f | catggtcctgctggagttcgtg |
| 9B3 | THB-seq-f-9B3 | ttaccggatacctgtcc |
| 9B4 | THB-seq-f-9B4 | aggatcttcacctagatcc |
| 9B5 | THB-seq-f-9B5 | actcatggttatggcag |
| 9B6 | THB-seq-f-9B6 | tatggtgcactctcagtac |
| 9B7 | THB-seq-f-9B7 | taaacctgtgattcctctg |
| 9B8 | THB-seq-f-9B8 | agatcctgcatataagc |
| 9B9 | THB-seq-f-9B9 | aacatcagaaggctgtag |
| 9B10 | THB-seq-f-9B10 | atcaagcagctccaggc |
| 9B11 | THB-seq-f-9B11 | atctcgacggtatcgatg |
| 9B12 | THB-seq-f-9B12 | ttcgataagtctctagcc |
| 9C4 | THB-seq-f-9C4 | catatgataatcaacctctgg |
| 9C5 | THB-seq-f-9C5 | ttaaacccgctgatcagc |
| 9C6 | THB-seq-f-9C6 | aacagggacttgaaagcg |
| 9C7 | THB-seq-f-9C7 | aacagagaggaatctttgcagc |
|  |  |  |
|  | **QL-cloning; Nested-PCR** |  |
| 8H1 | THB-8H1-for | TGAGTTGGATAGTTGTGG |
| 8D9 | THB-8D9-seq-f | catggtcctgctggagttcgtg |
| 8H2 | THB-8H2-rev | GGATTTATACAAGGAGGAGA |
| 5I3 | THB-5I3-fwd-QL-ZM96 | ATAATACGTCTCGCTAGCATGGGAGTGCGGGAGATCCTGCGGAACTGGCAGCGGTGGTGG |
| 5I4 | THB-5I4-rev-QL-ZM96 | ATATTCGTCTCCTCGAGCTAGTAGCCCTGCCGCACTCTGTTCACGATGGACAGCACGGCG |
|  |  |  |
|  | **qPCR** |  |
| 8H9 | THB-8H9-MN-rev-2 | GATGATGTTTTTGGTGGTG |
| 8I1 | THB-8I1-RF-rev-2 | AGTGAGCTTTTCGGATGT |
| 8G1 | THB-8G1-CDC4-rev | TCTCGCCAGTTGTGTACC |
| 8G3 | THB-8G3-HXB2-rev | GGTCCTCTCTGAATTCTA |
| 8G4 | THB-8G4-SF33-rev | CCCGGTAGTGTAGAGC |
| 8G5 | THB-8G5-V3-for | GGAAGGCATCATCATCAG |
| 8I2 | THB-8I2-V3-rev | CGATCTCGATGGATCTGTTC |
| 8I3 | THB-8I3-V3-Sonde (FAM-BHQ1) | TTTCACGTTGTTGGTCAGGTT |
|  | | |
