# Supplementary material for: A Mammalian Cell Based FACS-Panning Platform for the Selection of HIV-1 Envelopes for Vaccine Development
Source: PLoS One. 2014 Oct 3;9(10):e109196. doi: 10.1371/journal.pone.0109196 (PMC4184847; doi:10.1371/journal.pone.0109196)
Supplement: Table S2 — Plasmid constructs. A complete list of all plasmids that were used for this project. (DOC) [file pone.0109196.s008.doc]

**Table S2. Plasmid constructs**

| **Name** |  |
| --- | --- |
| pNL4-3 | pUC based vector, including the complete HIV-1 genome of the strain NY5/BRU (LAV-1), Accession number: AF324493.1 |
| pTN7-Stop | Lentiviral vector based on NL4-3, autologous Env ORF interrupted, includes *R.Luciferase* inserted into the Nef ORF |
| pTN pack | Lentiviral packaging construct in kind provided by K. Schilling |
| pWPXLd | Lentiviral vector, Addgene plasmid #12258, Addgene Inc., Cambridge, USA |
| pVSVG | Envelope of vesicular stomatitis virus cloned into pcDNA3.1(+). |
| pPCR-Script Amp | Commercially available vector, Agilent technologies # 211188 |
| pcDNA3.1(+) | Commercially available vector, Invitrogen # V790-20 |
| pcDNA3.1(+) QL | Derivative of pcDNA3.1(+), including a CcdB cloning cassette inserted at the MCS |
| pEGFP-C1 | Commercially available eGFP expressing vector, Clonetech, GenBank # U55763 |
| pEYFP-C1 | Commercially available eGFP expressing vector, Clonetech, Catalog # 6005-1 |
| pMACS LNGFR-IRES | Commercially available IRES vector, Miltenyi Biotec, Catalog # 130-091-887 |
| pQL9 | Lentiviral vector including GFP, IRES and a CcdB cloning cassette; 9.1 kb |
| pQL11 | Lentiviral vector including GFP TaVp2A and a CcdB cloning cassette; 8.2 kb |
